# Physical Activity and Excess Weight in Pregnancy Have Independent and Unique Effects on Delivery and Perinatal Outcomes

**DOI:** 10.1371/journal.pone.0094532

**Published:** 2014-04-10

**Authors:** Kelly L. Morgan, Muhammad A. Rahman, Rebecca A. Hill, Shang-Ming Zhou, Gunnar Bijlsma, Ashrafunnesa Khanom, Ronan A. Lyons, Sinead T. Brophy

**Affiliations:** 1 College of Medicine, Swansea University, Swansea, United Kingdom; 2 Department of Physiology, Radboud University Nijmegen Medical Centre, Nijmegen, The Netherlands; 3 Institute of Life Sciences, Swansea University, Swansea, United Kingdom; INRA, France

## Abstract

**Background:**

This study examines the effect of low daily physical activity levels and overweight/obesity in pregnancy on delivery and perinatal outcomes.

**Methods:**

A prospective cohort study combining manually collected postnatal notes with anonymised data linkage. A total of 466 women sampled from the Growing Up in Wales: Environments for Healthy Living study. Women completed a questionnaire and were included in the study if they had an available Body mass index (BMI) (collected at 12 weeks gestation from antenatal records) and/or a physical activity score during pregnancy (7-day Actigraph reading). The full statistical model included the following potential confounding factors: maternal age, parity and smoking status. Main outcome measures included induction rates, duration of labour, mode of delivery, infant health and duration of hospital stay.

**Findings:**

Mothers with lower physical activity levels were more likely to have an instrumental delivery (including forceps, ventouse and elective and emergency caesarean) in comparison to mothers with higher activity levels (adjusted OR:1.72(95%CI: 1.05 to 2.9)). Overweight/obese mothers were more likely to require an induction (adjusted OR:1.93 (95%CI 1.14 to 3.26), have a macrosomic baby (adjusted OR:1.96 (95%CI 1.08 to 3.56) and a longer hospital stay after delivery (adjusted OR:2.69 (95%CI 1.11 to 6.47).

**Conclusions:**

The type of delivery was associated with maternal physical activity level and not BMI. Perinatal outcomes (large for gestational age only) were determined by maternal BMI.

## Introduction

A normal birth, demonstrated by a vaginal delivery without the need for induction or instrumental intervention, promotes minimal intrusion and is often the anticipated mode of delivery amongst healthy expectant mothers [1], [2]. Suggestions of achieving normal births in 60% of healthy expectant mothers have been documented [3], however the definition of a ‘healthy woman’ is left open to ambiguity. Caesarean sections are associated with increasing risks for both mother [4], [5] and infant [6], [7], [8], [9] whilst the extra financial cost to the National Health Service has been estimated at £1,200 per pregnancy [10].

An extensive number of studies [11], [12], [13], [14] and reviews [15], [16] report the undesirable effects of increasing maternal BMI on the risk of caesarean section and operative vaginal deliveries. This increased risk has been shown to remain constant across women of varying ethnicities and parity [15]. Adverse outcomes for offspring have also been noted e.g. higher birth weight [17], [18], low APGAR scores [19], and foetal death [4] amongst pregnancies of overweight and obese women. However, not one of the aforementioned studies has accounted for the effects of maternal physical activity, a factor which could demonstrate an independent or residual confounding effect on the delivery and birth outcomes noted.

Exercising throughout pregnancy has been shown to have a positive effect on maternal psychological well-being [20], labour duration [21] and control of gestational weight gain [22], [23]. Other studies have failed to find any association between maternal exercise and birth outcomes other than enhancements in maternal fitness levels [24], [25]. One study examining the combined effects of maternal weight and self-reported physical activity levels concluded that physical activity levels did not counteract poor outcomes associated with higher maternal BMI and excessive weight gain [26].

With the number of overweight and obese women increasing [27] and physical activity levels generally declining throughout society [28], it is important to educate women on the birth related consequences. From a public health viewpoint it is important to ascertain which risk factor, overweight/obesity or low physical activity levels, has the greatest relative contribution to delivery and perinatal outcomes in order to inform future targeted policies and interventions. This prospective cohort study aimed to; 1) Examine the individual effects of maternal weight and physical activity on delivery and birth outcomes 2) Provide quantification of risks after controlling for potential confounding factors.

## Methods

This study formed part of the Growing Up in Wales: Environments for Healthy Living (EHL) study, a prospective birth cohort carried out in South Wales, United Kingdom. An outline of the study has previously been provided elsewhere [29]. The study was approved by the South East Wales Research Ethics Committee for Wales. Briefly, pregnant women are recruited face-to-face by study researchers or responded to study leaflets provided at local maternity hospitals. After providing written consent (form approved by Ethics Committee), women take part in a one-off home visit during pregnancy enabling anthropometric, demographic and questionnaire data to be collected. Questionnaires provide information on ethnicity, socioeconomic status, parental education, smoking status, alcohol consumption and previous pregnancies. Consent is also requested for researchers to access antenatal records, postnatal notes and routinely collected electronic medical records. Of the 661 women who have taken part in the EHL study prior to this investigation, 466 women were eligible to be included in the current study. Exclusion criteria were: multiple pregnancies, gestational diabetes, and missing exposure or outcome data.

### Exposure assessment

Pre-pregnancy Body mass index (kg/m^2^) (BMI) was defined using height and weight data recorded by midwives in the antenatal notes during early pregnancy (usually during week's 10–12 gestation). Women were divided into the following categories as recommended by the World Health Organisation [30]; normal weight (BMI between 18.5 kg/m^2^ and 24.9 kg/m^2^) and overweight (BMI between 25 kg/m^2^ and 29.9 kg/m^2^) and obese (BMI greater than 29.9 kg/m^2^).

Physical activity levels during pregnancy were monitored over 7 consecutive days with use of an accelerometer. Over the study period two types of accelerometer were distributed to study participants, both providing a measure of physical activity but employing different data extraction methods. A previous comparison between the accuracy of the two devices revealed virtually identical results when both devices were worn around the waist (Area Under the ROC Curve = 0.94) [31]. The details and study requirements of both accelerometers are outlined as follows:

1. A tri-axial Actigraph (GT3X) accelerometer [32] worn around the waist collected step counts on a 1-second epoch basis at a rate of 30 Hz. Due to the non-waterproof design of the unit, participants were informed to remove the belt when bathing, showering, and swimming. The accelerometer provided the total number of activity counts over the duration of 7 days which was used to estimate the participant's average daily activity count. Initially the raw data were stored and later converted into analysable data providing an average number of valid daily counts. Containing a ‘Wear time validation rule’ the software selected only days with a minimum of 8 hours wear.
2. A tri-axial GENEA accelerometer [33] worn on the non-dominant wrist collected seismic movements on a 1-second epoch basis at a rate of 100 Hz. Data provided by the GENEA accelerometer was coded using a previously validated method for ascertaining wear and non-wear time [34], [35]. The intensity of activity (defined as the ‘non sedentary SVM’) was used for physical activity classification purposes.

For the analyses presented in this paper women were ranked in order of activity score and dichotomised into two groups; ‘low activity’ if they were below and ‘high activity’ if they were at or above the 50^th^ percentile (using average daily counts (Actigraph accelerometer) or non sedentary Standard Vector Magnitude (SVM) (GENEA accelerometer)). ‘Data from the Actigraph accelerometer showed a reverse J distribution whereas GENEA accelerometer data presented a normal distribution.’

### Outcome measures

Accessing both hospital and electronic based health records provided information on delivery and perinatal outcomes. Manually collected postnatal notes were used as the main source of data but in the case of missing notes, an alternative data source, the Secure Anonymised Information Linkage (SAIL) databank, was used to access electronically-held routine birth outcomes [36]. The SAIL databank provides access to a wide range of patient data across large datasets. For the purpose of this study birth data regarding the infant was gathered from the National Community Child Health Database (NCCHD) and data concerning the delivery and the mother's health status was gathered from General Practice (GP) records.

Delivery variables collected were; Induction of labour, mode of delivery (vaginal, assisted (ventouse or forceps), elective caesarean section or emergency caesarean section), prolonged first (greater than 10.5 hours) [37] and second stage (more than 2 hours) [38] of labour and duration of hospital stay.

Perinatal outcomes were; Small for gestational age (SGA) (birth weight below the 10^th^ percentile for gestational age), large for gestational age (LGA) (birth weight above the 90^th^ percentile for gestational age) and low Apgar score (of 7 or less at 5 minutes). Gestational age was ascertained from the recorded estimated due date, which was determined by ultrasound scan and recorded in the antenatal records.

Throughout adjusted analyses potential confounders included maternal age, parity, smoking during pregnancy, alcohol consumption during pregnancy, maternal education and socioeconomic status.

### Data analysis

All statistical analyses were performed using STATA version 12.1 (STATA, Texas, USA). Differences in demographic characteristics and clinical outcomes between the two sub-groups (both BMI and physical activity) are expressed as number (percentage) and confidence intervals with a *P* value <0.05 implying statistical significance. Univariate and multivariate logistic regression analyses were performed reporting odds rations (OR), adjusted OR and 95% confidence intervals (CI). Each confounder (maternal age, smoking status, parity, education and income) was analysed in isolation and grouped to other confounders through multiple logistic regression analyses. Significant findings were also stratified by nulliparous and multiparous women to ascertain whether parity affected the results.

Bland-Altman plots, also known as Tukey mean-difference plots [39] were created in order to quantify the level of agreement between birth weights and gestational ages collected either manually or from electronic records. This method was designed to provide a visual comparison of the difference between two methods (y axes) which measure the same outcome throughout a range of values (mean value for the two methodologies, x axes). Providing a useful overview of data ranges and levels of concordance between methods, the Bland-Altman method has previously been shown as a more reliable approach than using the 95% limits agreement [40]. A range of agreement was defined as mean bias ±2 SD.

## Results

**Figure 1:**
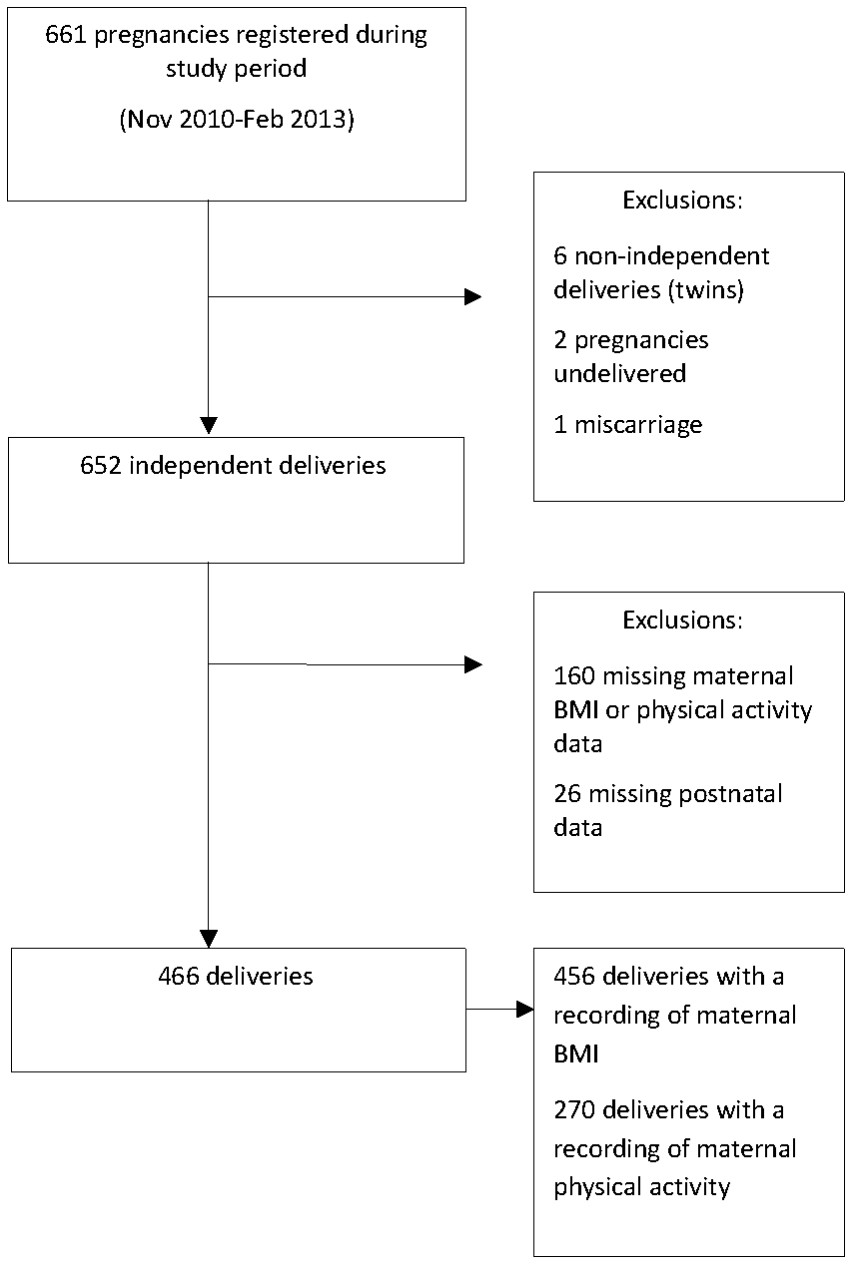
Number of singleton pregnancies throughout the study period after exclusions.

Formation of the study cohort is shown in figure 1. Women with multiple pregnancies, a non-live foetus and incomplete data were excluded. A total sample of 466 eligible women was included within this study of which 456 women had pre-pregnancy BMI data and 270 provided physical activity data. Amongst the pre-pregnancy BMI analyses, 212 (46.5%) women were assigned to the overweight/obese category, comprising of 148 (69.8%) overweight women and 64 (30.2%) obese women (BMI greater than 29.9 kg/m^2^).

Table 1 provides characteristics of the study population and a breakdown for categories of pre-pregnancy BMI and maternal activity levels. Amongst the whole study sample, available ethnicity data revealed over 90% of the population as Caucasian. Women were predominantly in the third (final) trimester during data collection (37% in the second trimester and 59% in the third trimester). Just under half (47.4%) of the study population were nulliparous. The majority of women reported some form of previous qualifications (92.5%); 56.7% had a household salary (before tax) above £25,000; one fifth (21.3%) of women reported smoking (22.2% of women smoked 5–10 cigarettes a day and 33.3% reported 10 cigarettes or more); and nearly 40% reported consuming alcohol during pregnancy.

**Table 1:** Demographics and characteristics of whole study population, categories of pre-pregnancy BMI and physical activity.

	Overall	Normal weight	Overweight/obese	Low activity	High activity
Characteristics	n (%)	n (%)	n (%)	n (%)	n (%)
N	466 (100%)	244 (53.5)	212 (46.5)	144 (50.4)	126 (49.6)
Caucasian	379 (90.5)	202 (90.2)	169 (90.4)	113 (91.9)	114 (96.6)
Gestation at visit					
1st trimester	7 (1.6)	4 (1.9)	1 (0.6)	1 (0.8)	2 (1.6)
2nd trimester	190 (43.6)	102 (40.3)	69 (38.8)	58 (43.9)	53 (44.2)
3rd trimester	239 (54.8)	101 (48.8)	108 (60.7)	73 (55.3)	65 (54.2)
Maternal age (year)					
<20	19 (4.3)	12 (5.3)	6 (3)	6 (4.5)	3 (2.5)
20–24	88 (20.1)	46 (20.2)	37 (18.4)	42 (31.6)	17 (14.2)
25–29	120 (27.3)	65 (28.5)	54 (26.9)	34 (25.6)	32 (26.7)
30–34	79 (18)	45 (19.7)	33 (16.4)	20 (15.0)	25 (20.8)
35–39	114 (26)	49 (21.5)	65 (32.3)	25 (18.8)	33 (27.5)
≥40	19 (4.3)	11 (4.8)	6 (3)	6 (4.5)	10 (8.3)
Parity					
0	221 (47.4)	125 (53.9)	73 (36.9)	77 (53.5)	58 (46.1)
1	150 (32.2)	67 (28.9)	77 (38.9)	35 (24.3)	45 (35.7)
2	66 (14.2)	30 (12.9)	30 (15.2)	26 (18.1)	15 (11.9)
≥3	29 (6.2)	10 (4.3)	18 (9.1)	6 (4.1)	8 (6.3)
Maternal education					
None	32 (7.5)	18 (8)	13 (6.7)	9 (7.0)	4 (3.4)
Trade	13 (3.0)	3 (1.3)	10 (5.2)	6 (4.7)	1 (0.8)
School attainment	116 (27.2)	66 (29.2)	48 (24.7)	39 (30.2)	27 (22.9)
Higher	181 (42.4)	101 (44.7)	78 (40.2)	48 (37.2)	68 (57.6)
Other qualification	85 (19.9)	38 (16.8)	45 (23.2)	27 (20.9)	18 (15.3)
Household salary (£)					
0–9,999	43 (11.6)	19 (10.2)	25 (14.1)	14 (13)	10 (9.5)
10,000–14,999	39 (10.6)	20 (10.8)	19 (10.7)	13 (12.0)	9 (8.6)
15,000–24,999	60 (16.3)	32 (17.2)	27 (15.3)	17 (15.7)	14 (13.3)
25,000–34,999	44 (11.9)	21 (11.3)	22 (12.4)	16 (14.8)	8 (7.6)
35,000+	165 (44.8)	86 (46.2)	76 (43)	44 (40.8)	59 (56.2)
Prefer not to answer	17 (4.8)	8 (4.3)	8 (4.5)	4 (3.7)	5 (4.8)
Maternal smoking					
Yes	90 (21.3)	53 (23.6)	32 (17.1)	31 (23.9)	21 (17.7)
No	332 (78.7)	172 (76.4)	155 (82.9)	99 (76.1)	98 (82.3)
Alcohol consumption					
Yes	259 (61.1)	80 (35.2)	79 (41.8)	44 (34.9)	56 (47.1)
No	165 (38.9)	147 (64.8)	110 (58.2)	84 (65.1)	63 (52.9)
**Delivery outcomes**					
Birth weight (kg)®	3.41±0.52	3.34±0.5	3.51±0.5	3.4±0.5	3.5±0.4
Gestational length (wks)®	39.5 ±1.4	39.5±1.6	39.6±1.5	39.3±1.7	39.7±1.4

* Values are presented as mean±SD.

Overweight/obese women were similar in most characteristics to normal weight women with the exception that overweight/obese women were less likely to be primipara (OR: 2.0 (95%CI:1.4 to 2.9)). Again similar proportions were shown when comparing characteristics of women of low and high activity levels whilst a greater number of women (borderline significance) with high levels of activity reported alcohol consumption (p = 0.05). On average women wore the Actigraph accelerometer for 4 days and the GENEA accelerometer for 5 days (of a requested 7 days). When comparing adherence to wearing an accelerometer between the two groups, high activity women wore the accelerometer for slightly longer (3 days v 4.3 days, p = 0.001).

Women excluded due to missing data (an outcome variable and/or birth details) presented similar characteristics to study participants. There was no difference in age (29.3 years (missing data) compared to 30.7 years (study group) or parity (45% compared to 47% first child, respectively). However, more of the study group reported smoking (21.3% compared to 15%) and consuming alcohol (38.9% compared to 27.5%).

Bland-Altman testing revealed that birth weight and gestational age were consistently provided by both the manually collected and electronic data demonstrating no significant difference (see Figure S1 and S2). For birth weight the 95% limits of agreement between the two methods ranged from −0.31 to 0.23 (kgs) with a mean difference of −0.02. Gestational age showed that the 95% limits of agreement ranged from −1.99 to 2.05 (weeks) for gestational age with a mean difference of 0.03. For both measures 1.6% of values were shown to be outside the limits of agreement.

### Delivery outcomes for total cohort

Of the total study cohort, 44% of women had a normal delivery (i.e. no induction or instrumental intervention), 23% had a caesarean section (of which 42% were an emergency) and 16% required some form of instrumental intervention. The mean (±SD) birth weight of offspring was 3.4 (0.5) of which 1.7% were SGA and 13% LGA. The mean (±SD) gestational age was 39.5 (1.6) and 1.5% of offspring were delivered preterm.

### Results for physical activity and delivery

Women with low activity levels had higher rates of forceps/ventouse deliveries (26 v 12.7%), and elective (21.5 v 15.4%) and emergency (14.9 v 8.7%) caesarean sections (Table 2). This finding remained significant after controlling for maternal age, parity and LGA (adjusted OR:1.72(95%CI: 1.05 to 2.9)). No significant associations between the level of physical activity and rates of induction, prolonged stages 1 and 2 or longer hospital stays after delivery were found.

**Table 2:** Unadjusted odds ratio for delivery and birth outcomes among pregnancies of low or high activity.

Outcome	Low Activity	High Activity	Unadjusted OR*	Adjusted OR
	n (%)	n (%)	(95% CI)	(95% CI)
	144 (53.3)	126 (46.7)		
Induction of labour	25 (26.9)	29 (31.9)	1.27 (0.67, 2.40)	
Instrumental delivery	20 (26)	10 (12.7)	0.41 (0.18, 0.95)*	
Caesarean delivery	45 (36.9)	26 (24.8)	0.56 (0.32, 1.0)*	1.72(1.05, 2.9)α
Length of stage 1>10.5 hours	6 (14.6)	8 (12.9)	0.86 (0.28, 2.70)	
Length of stage 2>2 hours	12 (16.7)	16 (24.2)	1.6 (0.69, 3.70)	
24 hours hospitalisation	19 (26.7)	25 (37.9)	1.44 (0.70, 3.0)	
48 hrs hospitalisation	20 (31.3)	22 (33.3)	1.1 (0.5, 2.3)	
>48 hrs hospitalisation	25 (39.1)	19 (28.7)	0.6 (0.3, 1.31)	
Delivery before 37 weeks	7 (5.26)	4 (3.0)	0.55 (0.16, 1.94)	
Delivery at 42 weeks	8 (6.0)	7 (5.2)	0.86 (0.30, 2.45)	
Small for gestational age	2 (1.5)	0		
Macrosomia	19 (13.2)	20 (15.9)	1.24 (0.63, 2.44)	
5 minute Apgar below 7	2 (1.0)	2 (1.5)	0.99 (0.14, 7.15)	

* Indicates that P<0.05

α Adjusted analysis encompass instrumental deliveries and caesarean section. Analyses controlled for the following variables: maternal age, parity and gestational age.

### Results for pre-pregnancy BMI and delivery

Overweight and obese women had an increased risk of induction (adjusted OR:1.92 (95%CI 1.14 to 3.26)) and a longer hospital stay (adjusted OR:2.68 (95%CI 1.11 to 6.47)) (Table 3). These findings remained significant after adjusting for post-term delivery, birth weight, parity, maternal smoking and induction of labour. There was no association between caesarean delivery and maternal BMI even after sub-grouping elective and emergency caesareans (OR:0.66 (95%CI 0.26 to 1.68)). Whilst similar rates of emergency caesareans (9.1% v 10.5%) were evident, there was a trend for higher rates of elective (10.2 v 16.3%) caesarean sections among overweight/obese women (p = 0.051).

**Table 3:** Unadjusted odds ratio for delivery and birth outcomes among normal- and overweight/obese pregnancies.

Outcome	Normal weight	Overweight/obese	Unadjusted OR*	Adjusted OR
	n (%)	n (%)	(95% CI)	(95% CI)
Induction of labour	41 (24.9)	52 (35.1)	1.64 (1.01, 2.67)*	1.92 (1.14, 3.26)∧
Instrumental delivery	18 (10.2)	26 (16.3)	1.70 (0.89, 3.24)	
Caesarean delivery	36 (20.5)	42 (26.3)	1.38 (0.83, 2.3)	
Length of stage 1>10.5 hours	12 (12)	8 (9.3)	0.74 (0.29, 1.91)	
Length of stage 2>2 hours	20 (17.1)	23 (21.5)	1.33 (0.68, 2.59)	
>24 hours hospitalized after delivery	40 (40.8)	56 (59)	2.08 (1.17, 3.70)*	2.68 (1.11, 6.47)¥
Delivery before 37 weeks	10 (4.1)	6 (2.7)	0.69 (0.24, 1.92)	
Delivery at 42 weeks	10 (4.1)	18 (8.6)	2.18 (0.99, 4.84)	
Small for gestational age	5 (2.1)	2 (1.0)	0.45 (0.09, 2.36)	
Macrosomia	22 (9.2)	35 (16.6)	1.97 (1.12, 3.48)*	1.96 (1.08, 3.56)∞
5 minute Apgar below 7	4 (1.7)	2 (1.0)	0.56 (0.10, 3.06)	

Analyses controlled for the following variables: ∧post-term birth, birth weight and parity, ¥Post-term birth, birth weight, parity, induction, ∞Gestational length, parity, smoking status.

### Results for physical activity, BMI and perinatal outcomes

Comparing perinatal outcomes between women of low and high activity levels revealed no significant differences. Similarly maternal pre-pregnancy BMI was not shown to impact on Apgar scores at 1 or 5 minutes, small for gestational offspring or gestational length. Overweight/obese women were however at an increased risk of delivering a LGA baby (adjusted OR:1.96 (95%CI 1.08 to 3.56)) even after adjusting for gestation, parity and maternal smoking. It is important to note that BMI and physical activity were not significantly associated in our study (mean BMI values; 25.9 kg/m^2^ in the low active group and 24.8 kg/m^2^ in the high active group, p = 0.14).

## Discussion

This study sought to assess the independent effects of maternal physical activity and pre-pregnancy BMI on birth delivery and perinatal outcomes. We found that physical activity levels influenced the type of delivery, with lower activity levels increasing the risk of an instrumental delivery. Overweight/obesity showed a trend for increasing numbers of elective caesarean sections and was significantly associated with greater risk of induction, longer hospital stay and delivery of a LGA infant. In contrast to most studies, we did not include maternal BMI (and vice versa physical activity) as a confounder when analysing the effects of physical activity on delivery and perinatal outcomes. We felt this was not necessary as; 1) significant outcomes differed for both physical activity levels and maternal BMI, 2) physical activity and maternal BMI were not significantly related in our study and 3) maternal BMI or physical activity can potentially act as mediating variables for many outcomes.

### Limitations and strengths

It is important to acknowledge a few limitations within our study design. Firstly, data collection took place shortly after the recruitment of a participant; consequently women were at varying time points in pregnancy when physical activity data were collected. As reported women were predominantly in the third trimester during data collection therefore our findings reflect activity levels at a time point where women may find it difficult to continue with normal activity routines. Given the one-off data collection design (i.e. data were collected at only one time point during pregnancy) we were unable to comment on changes in activity levels as pregnancy progressed or the impact of employment-related physical activity. However, it is important to note that gestation at time of data collection was not a confounding factor amongst our analyses.

Secondly, there were evident issues with our physical activity data. First, using two types of accelerometry data limited our ability to look at physical activity levels on a continuous scale and instead cut-offs at the 50 percentile were used. Second, despite providing participants with an accelerometer for 7 days in total, on average women wore the device for 4 (Actigraph accelerometer) or 5 days (GENEA accelerometer) of which we did not differentiate between week days or weekends. Third, physical activity data were not available for 42% of participants which may reflect participant adherence to wearing an accelerometer, accelerometer availability, and/or missing data due to faulty units. Lastly, we cannot exclude the effects of information bias or residual confounding due to unmeasured factors (e.g. genetic factors, paternal factors, maternal diet, and gestational weight gain).

Several strengths of this study are evident including the use of objective physical activity and clinically recorded pre-pregnancy BMI data. First, our findings offer a more accurate insight into the observed effects when compared to studies using observational [12], self-report BMI [5], [41], [42] and self-reported physical activity [22], [43], [44], [45], [46], [47]. We are also able to avoid the bias associated with self-reported data. Second, adopting a prospective design our study has been able to consider and adjust for numerous confounding factors collected through field research. Third, extracting study outcomes directly from postnatal medical records and routine data provided completeness of data and lack of recall bias.

### Interpretation

Currently there is fairly inconsistent evidence on the relation between maternal activity levels and mode of delivery. In agreement with our findings, studies based on self-reported activity [23] and structured exercise programmes [43], [48] have also demonstrated lower rates of caesarean section deliveries amongst more active women. A proposed explanation for this finding is the ability of physically active women to cope with the demands of birth through maintained or enhanced fitness [23]. It may also be speculated that women with lower activity levels lead unhealthier lifestyles with regards to diet and weight control [49]. However, contradictory to our findings, other studies have failed to find any association with delivery type when considering energy expenditure [50] and self-reported activity [47], [51]. Magann and colleagues [50] derived energy expenditure through participant's completing a questionnaire and a daily record of activity/work. Despite finding no association with delivery mode, other associations were observed between activity levels and delivery outcomes. Importantly however, the authors noted attenuation towards the null hypothesis following adjustments for confounding factors.

We did not find any other associations between physical activity levels and delivery outcomes. To our knowledge there are limited studies available using objective measures of physical activity. In the present study we extend previous findings through use of objective physical activity measures over a 4–5 day period. Although we cannot comment on which types of physical activity were carried out, our findings provide supportive evidence for higher levels of daily activity positively impacting upon delivery mode. Given our observational study design however, randomized clinical trials are needed to further clarify the cause and effect of instrumental deliveries whilst also considering the effects of maternal co-morbidities.

Previous studies investigating the effects of overweight and obesity on delivery outcomes have relied on self reported data or not accounted for a wide range of confounding factors. Our study findings are consistent with previous research [52], [53] reporting higher rates of induction and a longer length of maternal stay in hospital amongst overweight/obese women. Specifically we found that overweight/obese women had 1.92 times the risk of requiring an induction than normal weight women. Jensen and colleagues [54] individually analysed the risks of induction amongst overweight and obese women reporting odds ratios of 1.5 and 3.2 respectively; however our sample sizes were too small to individually report odds ratios on each BMI category. Furthermore, despite reporting no significant effects of maternal pre-pregnancy BMI on delivery mode, we observed a trend for increasing rates of elective caesarean sections amongst overweight/obese women. Our ability to detect a trend whilst using a relatively small study population warrants further investigation. We did observe longer hospital stays amongst the overweight/obese women. This finding has been reported previously [13], [55] and potentially reflects increasing risks among overweight and obese women of; infective complications [56], postpartum haemorrhage and a greater need for antibiotic use [53], which were not assessed in this study.

Our lack of support for a relationship between physical activity and perinatal outcomes are consistent with previous studies which have also failed to find any effect on neonatal Apgar scores [24], [57], [58], [59], [60], [61], whilst the effects on birth weight remain uncertain. Conversely, our finding of an increasing risk of delivering a LGA infant amongst overweight and obese women is well established amongst the current literature, even amongst women exempt from diabetes [62]. In agreement, a comparatively sized prospective cohort reported higher levels of LGA amongst overweight women whilst finding no effect of physical activity levels during pregnancy on offspring size [63]. Utilising questionnaires, the authors gathered retrospective physical activity data defining women of ‘low activity’ as those reporting exercising for less than 1 hour per week. Physical activity levels before (but not during) pregnancy were associated with offspring size, and inactivity before pregnancy was an independent risk factor for delivering a LGA infant. Furthermore, a recent study [64] found no effect of maternal physical activity levels on offspring size after controlling for maternal pre-pregnancy BMI. In agreement with our findings, the authors concluded that maternal overweight and obesity were more influential on perinatal outcomes than maternal activity levels.

Weight gain and physical activity are both modifiable health behaviours which can be altered before and/or during pregnancy. Our findings show independent and unique effects of both maternal factors on delivery and perinatal outcomes. From an obstetric point of view increasing maternal physical activity levels will lead to a reduction in the number of instrumental and caesarean section deliveries. This may also provide an indirect route for targeting weight reduction or control amongst overweight or obese women.

## Supporting Information

Figure S1 **Bland Altman plot showing concordance of methods when collecting birth weight data.**
(DOCX)

Figure S2 **Bland Altman plot showing concordance of methods for collecting gestational age data.**
(DOCX)
