# Supplementary material for: Physical Activity and Excess Weight in Pregnancy Have Independent and Unique Effects on Delivery and Perinatal Outcomes
Source: PLoS One. 2014 Apr 10;9(4):e94532. doi: 10.1371/journal.pone.0094532 (PMC3983184; doi:10.1371/journal.pone.0094532)
Supplement: Figure S2 — Bland Altman plot showing concordance of methods for collecting gestational age data. (DOCX) [file pone.0094532.s002.docx]

**Figure S2:** Bland Altman plot showing concordance of methods for collecting gestational age data


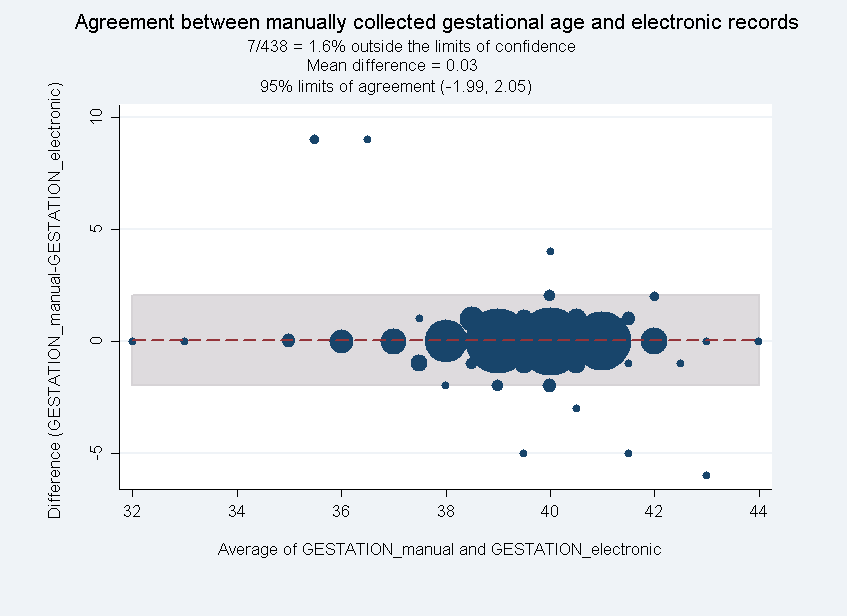


(wks)

(wks)
